# Supplementary material for: Patterns of Intron Gain and Loss in Fungi
Source: PLoS Biol. 2004 Nov 30;2(12):e422. doi: 10.1371/journal.pbio.0020422 (PMC532390; doi:10.1371/journal.pbio.0020422)
Supplement: Table S1 — Also available at http://genes.mit.edu/NielsenEtAl/. (4.3 MB ZIP). [file pbio.0020422.st001.zip › NielsenEtAl/html/603.html]

AN7517.1.NCU03793.1.MG07464.1.FG07378.1


```
 CLUSTAL W (1.82) Multiple Sequence Alignments - Introns Inserted


Sequence 1: NCU03793.1	449 aa
Sequence 2: MG07464.1	457 aa
Sequence 3: FG07378.1	445 aa
Sequence 4: AN7517.1	444 aa
Alignment Length: 483 aa
Number Identitical Residues: 90 aa
Alignment Score (without introns) 6331


MG07464.1 	---MATPNRIHIAP-ENTGLLKVS-----QNADAAKTVTELLQKDLE0TT----DQETSP
NCU03793.1	MSAEATPHRINITP-SNIGLWSKSPTIHSQPPTAYTLLSDLLQADMH~DHHVFFNKDGFH
FG07378.1 	---MATPFQIQVEP-QNSGLLGFK-----VGQSEASKVTDLLQKDLE0NHHVFFNESGFH
AN7517.1  	---MATARKVLLSPRSDTGVWSTG-----ITESAAKTASAALQEDLE~KHHVYFNDMGFH
          	    **. :: : * .: *:               .  :  ** *:.       :.    

MG07464.1 	K~K~IVHHLLSLYGTSAGPSSLKKAWEGNTSYQRPVQPTHASATPAQLADWAT-AKQYLC
NCU03793.1	N~H~IPHHLLALYGTSAPSEALQKAYDVNASYQRPTLPEHQEVVSDLIQNWRDDAAKYLG
FG07378.1 	D~H0LVHQVLTLYGTGATTDILTKAYDANKTYQLKAMKPKSDVIDRLEH--GSDWSEYLG
AN7517.1  	D1H~IAHQILTLYALGASPDEIQAAYDRNKTYQRKALPMHEDVVNGLHQ--KDKFKDALG
          	. : : *::*:**. .* .. :  *:: * :**  .   : ..          .  . * 

MG07464.1 	KEQYYPDFLAFFQREIETSS---VADVLNRYVFNEGDERADDMLIRLFGG~FVHPLIQLM
NCU03793.1	KEEHYPDFLAFFKREMDSRGEGKYEQVVNEYLF-AGTPEADNLLVRLYSG~FLHPLIQLM
FG07378.1 	KGRNYATFFRFFQDEIERIG---WQDTLKEYLF-KDDARGRDMQSRLFAG~VLHPLIQLL
AN7517.1  	KEKNYPDFLEFFQREIDKKG---VEAVLSEYLF-KQDENAESMMARLFGG1LLHPIIHLG
          	* . *. *: **: *::  .      .:..*:*     .. .:  **:.* .:**:*:* 

MG07464.1 	YGIEWNQPVIVAEGLAQACVHRDDLR-AFMLGAEKLSREQQKQGGDDDKMGSILS--LYE
NCU03793.1	YGLEWKQPAIVAEGLAQAAVHGDEIG-GYLLGAEQAAKASSKP---DDGMGRVVEDLLER
FG07378.1 	YGMEWEQPMIIAAALAQTAVHRDDYH-EFLTSAAKKAESDNAP----PRMKTIPG--LYE
AN7517.1  	FGIEFNQPAIIAEALAQTAIHEDWTGPRFLFPAEQVAGGIGKP-----GRKTMLQ--LLE
          	:*:*::** *:* .***:.:* *   . ::  * : :               :    * .

MG07464.1 	AIPRDEKISSAAREEDANKMRDGVLSRAGDEMAALAARVRVRPEELHERTVEMFNAAV~Y
NCU03793.1	GVRGNRKLAEAARMSDANKVRDGVLKRAKEEAIQLAAKVKVGKEELQERTAEMYDSAV~F
FG07378.1 	EVVKNEKLVKSSHWEDSNRVFDGVLAREKEEMINLAARVRIDKNELDERAAEMVHNAA~F
AN7517.1  	EAQENTKLTNSVRFEDGNKLRDGVLKRAGKDMIELAAQYTVSEEQMAEKYAEIVDVSV1Y
          	    : *: .: : .*.*:: **** *  .:   ***:  :  ::: *: .*: . :. :

MG07464.1 	VAASAAVARP-VKHAKFDFFLI2HHVNVSPILLSINAASWIPTAAKVRLLEWKIRIDLVQ
NCU03793.1	MASAAALVKRNTKLPRFDFFLM2HHVTSAPFFTTVNAQDWISLDTKVRLLEWKIRMDLVQ
FG07378.1 	VVAGAAFHPP--YHPRFDFILM2HHTTSTPFFLVLNKQSWIPASTKARLLENKIRMDILQ
AN7517.1  	FTAAS--QRP-TKSVKFDFFYI~HTVNSSIFFAKILVLPFLHTATKLRLLEMKGRMDLFM
          	..:.:          :***: : * .. : ::  :    ::   :* **** * *:*:. 

MG07464.1 	YAARAVPPLSLERIAAYQPKPKDDRFNTAAAAGSSSNVGD~LVSR-LHDLDDDGHAIKLV
NCU03793.1	YAARGAPELSLDAITSYQPKEKK----------TSGTPEE1IISN-LHFFEDDGHSIKLA
FG07378.1 	YIARGSPALRVDMLRDYKPKDGDK---------LVEKPED~LFPR-IHKIIDDGHTVKLA
AN7517.1  	YVSRNAPQLYLGEVTKYP---------------INQTWES~IIKQSVQHTHDDGHLVKLI
          	* :*  * * :  :  *                   .  . :. .:::   **** :** 

MG07464.1 	RAAAICQQVSEPLEDKLGDRLPLRGKEIWNKVHHLI~VDAAEGP----GPRWVRTTGLES
NCU03793.1	RAARICQLYYQTRDD-----------DYWNKVYHLI~VDSVLAP----GPNWVRSCGFDE
FG07378.1 	RALMLAQRVTEPYQDRDWVRIK---DGEWLKAVYVL~MDANEEADSQEGTMWVRSAGFDE
AN7517.1  	RALKHGEAVCRTYEGQGREKGLKITGDSWLRIGNMV1VVIDSVHGRGEEKMWVRSTGFDE
          	**    :   .. :.             * :   :: :      .  .   ***: *::.

MG07464.1 	AWK0DVPDAPKL
NCU03793.1	AWE~DIPNVE--
FG07378.1 	AWE~EIPKAKM-
AN7517.1  	AWD~AVEDRARL
          	**.  : .
```
